# Multiplexed editing of a begomovirus genome restricts escape mutant formation and disease development

**DOI:** 10.1371/journal.pone.0223765

**Published:** 2019-10-23

**Authors:** Anirban Roy, Ying Zhai, Jessica Ortiz, Michael Neff, Bikash Mandal, Sunil Kumar Mukherjee, Hanu R. Pappu

**Affiliations:** 1 Department of Plant Pathology, Washington State University, Pullman, WA, United States of America; 2 Advanced Centre for Plant Virology, Division of Plant Pathology, Indian Agricultural Research Institute, New Delhi, India; 3 Department of Crop and Soil Sciences, Washington State University, Pullman, WA, United States of America; Oklahoma State University, UNITED STATES

## Abstract

Whitefly-transmitted begomoviruses cause serious damage to many economically important food, feed, and fiber crops. Numerous vegetable crops are severely affected and chilli leaf curl virus (ChiLCV) is the most dominant and widely distributed begomovirus in chilli (*Capsicum annuum*) throughout the Indian subcontinent. Recently, CRISPR-Cas9 technology was used as a means to reduce geminivirus replication in infected plants. However, this approach was shown to have certain limitations such as the evolution of escape mutants. In this study, we used a novel, multiplexed guide RNA (gRNA) based CRISPR-Cas9 approach that targets the viral genome at two or more sites simultaneously. This tactic was effective in eliminating the ChiLCV genome without recurrence of functional escape mutants. Six individual gRNA spacer sequences were designed from the ChiLCV genome and in vitro assays confirmed the cleavage behaviour of these spacer sequences. Multiplexed gRNA expression clones, based on combinations of the above-mentioned spacer sequences, were developed. A total of nine-duplex and two-triplex CRISPR-Cas9 constructs were made. The efficacy of these constructs was tested for inhibition of ChiLCV infection in *Nicotiana benthamiana*. Results indicated that all the constructs caused a significant reduction in viral DNA accumulation. In particular, three constructs (gRNA5+4, gRNA5+2 and gRNA1+2) were most effective in reducing the viral titer and symptoms. T7E1 assay and sequencing of the targeted viral genome did not detect any escape mutants. The multiplexed genome-editing technique could be an effective way to trigger a high level of resistance against begemoviruses. To our knowledge, this is the first report of demonstrating the effectiveness of a multiplexed gRNA-based plant virus genome editing to minimize and eliminate escape mutant formation.

## Introduction

Begomoviruses (family *Geminiviridae*) are circular ssDNA viruses with genomes of 2.7 (monopartite) or 5.4 kb (bipartite). Begomoviruses are transmitted by whiteflies (*Bemisia tabaci*) and cause serious damage to numerous economically important pulse, vegetable and fiber crops in tropical and subtropical regions of the world [1–3]. Among the different vegetable crops, chilli (*Capsicum annuum*) suffers from a leaf curl disease caused by different begomoviruses [4]. Chilli leaf curl virus (ChiLCV) is the most predominant virus in the Indian subcontinent causing leaf curl in chilli and often accounts for 100% yield loss [5].

Begomoviruses have one or two genomic components (DNA-A and DNA-B). Majority of begomoviruses in the Old World have monopartite genomes containing one DNA component, similar to the DNA-A of bipartite begomoviruses. Often, monopartite begomoviruses are associated with a betasatellite that enhances virus virulence [6]. The genome of monopartite begomoviruses has six open reading frames (ORFs), two (V1 and V2) in viral sense and four (C1, C2, C3 and C4) in complementary sense. All proteins encoded by monopartite begomoviruses are multifunctional. V1, codes for a coat protein (CP), is involved in encapsidation of the genome into geminate particles, insect transmission and long distant movement. V2 is believed to play a role in cell-to-cell movement and suppression of RNA silencing of the host. C1, codes for a replication initiator protein (Rep), is involved in viral DNA replication. C2, codes for a transcription activator protein (TrAP), activates viral DNA transcription and is also a host RNA silencing suppressor. C3, codes for a replication enhancer protein (REn), plays a role in enhancing viral DNA replication, and the protein coded by C4 is known to be a silencing suppressor [7–9]. Besides these ORFs, the begomovirus genome has a non-coding intergenic region (IR), which possesses a bidirectional promoter and a conserved nona-nucleotide sequence (TAATATTAC), recognized by the C1 protein, acting as the origin of replication. The nona-nucleotide sequence is recognized by the AC1/C1 protein, and it serves as the origin of replication.

Management of viral diseases, especially those caused by begomoviruses, is challenging and expensive. Some of the approaches include control of the whitefly vector through insecticides, and resistant cultivars [10–13]. In tomato, host plant resistance to begomoviruses mainly was based on the use of *Ty* genes [14]. Another approach is the use of RNAi-based transgenic technology against bean golden mosaic virus (BGMV) in common bean in Brazil [15]. These approaches are specific to a begomovirus or its close relatives. However, in a field situation, multiple begomovirus species are known to cause the same disease. Hence, it is necessary to develop efficient and durable tactics for conferring broad spectrum resistance against begomoviruses.

CRISPR-Cas9 based genome editing shows great promise to reduce geminiviral infection [16]. The CRISPR-Cas9 system is an antiviral defence mechanism in bacteria that protects against invading DNA viruses and/or plasmids [17]. Recently, it was used for target-specific mutagenesis by inducing double strand breaks (DSBs) in DNA at a specific location in different organisms [18]. Such DSBs are either repaired by the inaccurate non-homologous end-joining (NHEJ) repair machinery or by precise homology-directed repair (HDR) [19,20]. The unrepaired DSBs are finally degraded. In most cases, mutated virus variants generated through NHEJ cannot replicate and move systemically due to the induced frameshift in the ORFs leading to a non-functional translated product. The wide ranging application of genome editing in plant virus management was discussed in recent reviews [21,22].

Over the last few years, the use of CRISPR-Cas9 editing for the management of ssDNA and dsDNA viruses was reported [23–28]. Examples include bean yellow dwarf virus (BeYDV, genus *Mastrevirus*) in *Nicotiana benthamiana* [25], beet severe curly top virus (BSCTV, genus *Curtovirus*) in *Arabidopsis thaliana* and in *N*. *benthamiana* [26] and tomato yellow leaf curl virus (TYLCV, genus *Begomovirus*) in *Nicotiana benthamiana* [23]. The gRNA-Cas9 system was also successful in simultaneously targeting three geminiviruses: beet curly top virus (BCTV) (genus *Curtovirus*), merremia mosaic virus (MeMV) and TYLCV (genus *Begomovirus*) when gRNAs specific for the IR sequence of each virus were used [23]. Ali et al. [24] demonstrated the efficiency of genome editing in cotton leaf curl Kokhran virus (CLCuKoV) and MeMV and evaluated the efficiency of the CRISPR-Cas9 machinery for targeting different coding and non-coding sequences of geminivirus genomes. However, they indicated that if gRNA is designed from coding regions, then such system generates geminivirus mutants that may evade CRISPR-Cas9 system. Such concern of development of functional mutant virus (escape mutant) after NHEJ got more emphasis when recently, Mehta et al. [28] applied the CRISPR–Cas approach in cassava. Unexpectedly, they found that the CRISPR-Cas9 system was insufficient to confer resistance to cassava against African cassava mosaic virus (ACMV) due to development of escape mutants, which may give rise to expansion of new virus variants. In this context, Rybicky [29] critically analysed their concern about such “CRISPR-induced virus evolution” and indicated that the study by Mehta et al. [28] missed an opportunity to fully investigate the potential of Cas9 for engineering resistance to plant viruses by focusing too narrowly on just one sgRNA, one Cas9, and transgenic cassava. He suggested that multiplexing of gRNA targeting different viral genomic region and proper expression of Cas9 could be the alternatives to achieving the goal. Targeting multiple regions of the viral genome simultaneously to restrict the formation of escape mutants has also been suggested earlier [30]. To investigate such multiplexing approach, we used a multiplexed gRNA-based strategy for simultaneously targeting different genomic regions of the ChiLCV genome and show that certain combinations of multiplexed-gRNAs provided a high degree of resistance to ChiLCV and restricted the generation of escape mutants. This approach could be broadly applicable to other geminiviruses.

## Materials and methods

### Plant materials and infectious virus construct

*N*. *benthamiana*, was used to assess the efficacy of multiplexed gRNA-based CRISPR-Cas9 modules to inhibit ChiLCV. An infectious clone of ChiLCV isolate (Accession number MK882926), generated by cloning a partial tandem repeat (PTR) of the viral genome. Briefly, first a complete viral genome was amplified by rolling circle amplification (RCA) using standard methodology followed by digestion of the RCA product using *Bam*HI [31]. The full length viral genome (ChiLCV-BamHI) was cloned in pUC18 vector and sequenced. A *BamH*I -*Eco*RI digested partial genomic fragment (1.1 kb) containing the origin of replication was cloned into pCAMBIA2300 vector. Further the *BamH*I digested full length genome was ligated in tandem orientation into the *BamH*I -*Eco*RI digested partial fragment cloned in pCAMBIA2300 and thus the PTR clone was obtained. The PTR clone, when co-agroinfiltrated with a croton yellow vein mosaic betasatellite to different chilli genotypes, produced severe leaf curl symptoms (unpublished). In *N*. *benthamiana*, the infectious clone alone caused typical leaf curl and stunting symptoms, and the symptom severity increased many fold in the presence of the betasatellite (unpublished). This infectious clone was used in the study.

### Selection of targeted regions and design of spacer sequences for editing the ChiLCV genome

**Fig 1:**
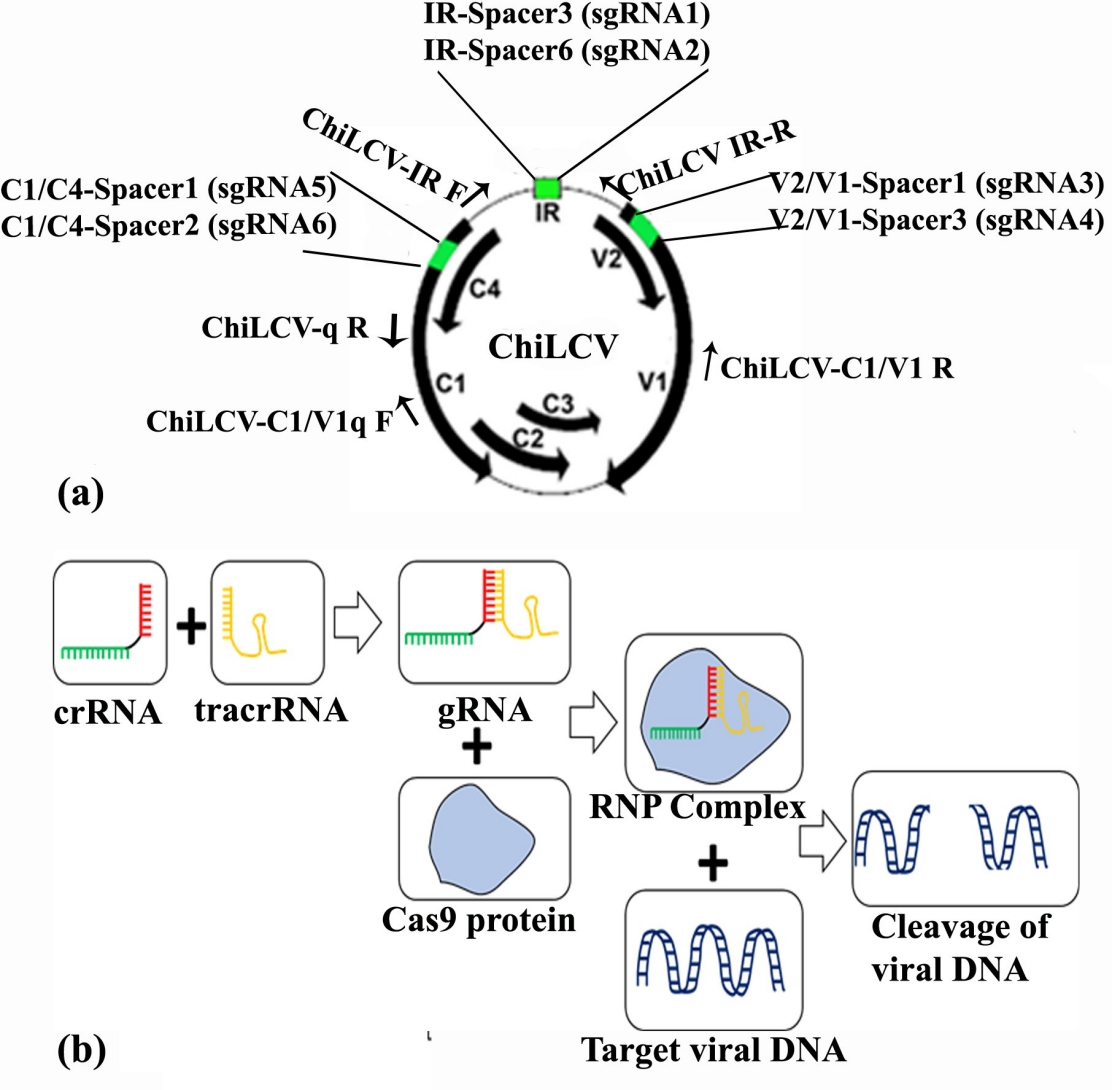
Position of different primers and spacer sequence in the chilli leaf curl virus (ChiLCV) genome and plan for in vitro cleavage assay. a) Three genomic regions (green color) of the ChiLCV were selected to identify the spacer, including i. Intergenic region (IR) with the origin of replication, ii. Overlapping regions between V2 and V1 genes (V2/V1) and iii. Overlapping regions between C1 and C4 genes (C1/C4); b) schematic representation of *in vitro* cleavage assay.

Three regions in the ChiLCV genome were selected for designing gRNAs. The three regions targeted are as follows: i. Intergenic region (IR) including the origin of replication, ii. Overlapping regions between V2 and V1 genes (referred to as V2/V1), and iii. Overlapping regions between C1 and C4 genes (C1/C4) (Fig 1A). The 20-nt spacer sequences were chosen based on the efficacy and specificity to the targeted region and the presence of an adjacent N-G-G sequence (PAM motif) (Table 1). All spacer sequences were analysed for possible off-targets in the *N*. *benthamiana* genome through a BLAST search. Spacer sequences with relatively higher GC content and no detectable off-targets were chosen.

**Table 1:** Details of spacer sequences selected from chilli leaf curl virus (ChiLCV) genome and their final selection based on off-target analysis and GC%.

Genomic region	Nucleotide coordinate and spanning length	Spacer name	Seq	PAM	Off Target analysis (10 top hit)				GC%
					Position in the genome	Target match in NB genome	Seed seq match	PAM match	
IR region with origin of replication	1–145 nt, span: 145 nt and 2611–2762, span: 152 nt	IR-Spacer1	CAATCGGTACTCAACAAACT	TGG	2622–2641	no	9–20 match	No	40
		IR-Spacer2	AACAAACTTGGCTATGTAAT	CGG	2634–2653	no	11–21 match	No	30
		IR-Spacer3	*GCCATCCGCACTAATATTAC	CGG	2745–2762, 1–2	no	9–20 match	No	45
		IR-Spacer4	TGGCCGCGATTTTTTTACCG	TGG	7–26 nt	no	12–23 match	Yes	50
		IR-Spacer5	CTCATAGCTTAATTATTTCA	TGG	81–100	no	12–23 match	No	25
		IR-Spacer6	*CATGGTCCCCCCTATAAACT	TGG	99–118	no	12–21 match	No	50
Overlapping betwwen V2 and V1 ORFs (V2/V1)	306 nt– 502 nt, span: 197 nt	V2/V1-Spacer1	*CATTTCCACGCCCGCCTCGA	AGG	332–351	no	12–20 match	No	65
		V2/V1-Spacer2	CCCCACTGTCCGCGTCACAA	AGG	404–423	no	14–23 match	Yes	65
		V2/V1-Spacer3	*AGGCCAGAGCATGGGTGAAC	AGG	424–443	no	12–21 match	No	60
		V2/V1-Spacer4	ACAGGAAGCCCAGGATGTAC	AGG	454–473	no	10–20 match	No	55
Overlapping between C1 and C4 ORFs (C1/C4)	2160–2459 nt, span: 300 nt	C1/C4-Spacer1	*GGACCCTGAATTGATTGCCT	CGG	2185–2204	no	10–21 match	No	50
		C1/C4-Spacer2	*TAGCTGATCTTCCATCGACT	TGG	2241–2260	no	10–20 match	No	45
		C1/C4-Spacer3	CTTTTAGCTCCCTGAATGTT	CGG	2324–2343	no	9–20 match	No	40
		C1/C4-Spacer4	GGTCGAAGAATCTGTTGTTT	TGG	2379–2398	no	11–23 match	Yes	40
		C1/C4-Spacer5	ATTTACCTTCGAATTGTATG	AGG	2409–2428	no	12–23 match	Yes	30
		C1/C4-Spacer6	TGTATGAGGACGTGGAGATG	AGG	2423–2442	no	13–23 match	Yes	50

*Spacer sequences finally selected

### *In vitro* cleavage assay for determining the efficacy of gRNAs

To verify the efficacy of the gRNAs designed, the genomic region of ChiLCV containing all three target sites was amplified using primers ChiLCV-C1/V1q F and ChiLCV-C1/V1 R (Table 2). The locations of different primers in the ChiLCV genome are shown in Fig 1A. An *in vitro* cleavage assay of the viral target amplicon was performed using Alt-R® CRISPR-Cas9 system (Integrated DNA Technologies, Coralville, IA, USA) following the manufacturer’s protocol. Briefly, Alt-R CRISPR-Cas9 crRNAs were synthesized against all the selected spacer sequences designed from the ChiLCV genome. Equimolar amounts of the individual Alt-R CRISPR-Cas9 crRNA and universal Alt-R CRISPR-Cas9 tracrRNA were incubated for 5 min. at 95°C in a nuclease-free duplex buffer and the solution was allowed to cool down to room temperature to form the gRNA (Alt-R CRISPR-Cas9 crRNA:tracrRNA). The gRNA complex was then combined with Alt-R S/p Cas9 Nuclease 3NLS to form a ribonucleoprotein (RNP) complex (Fig 1B), which was then incubated with the viral target amplicon for 1 hr at 37°C. Post-cleavage fragments were analysed by agarose gel electrophoresis. The assay was performed with individual RNP complexes or with different duplex and triplex combinations.

**Table 2:** Details of primers used in the study.

Purpose	Primer combinations and sequence (5’-3’)	Amplification condition*		Expected amplicon size (kb)
		Annealing temperature (^0^C)	Extension time (Sec) at 72°C	
Amplification of all the target site for *in vitro* cleavage assay	ChiLCV-C1/V1q F (GGGCTAAGGTCGAGATGTCC)ChiLCV-C1/V1 R (CATCCATCCATATCTTCCCTAATAC)	59	45	1.5
Generating amplicon for T7E1 assay and sequencing	ChiLCV-C1/V1q F (GGGCTAAGGTCGAGATGTCC)ChiLCV IR-R (GTCGCTTGGACATAATTCTTAGC)	61.2	37	1.2
	ChiLCV-IR F (ATTGTATGAGGACGTGGAGATGAG) ChiLCV-C1/V1 R (CATCCATCCATATCTTCCCTAATAC)	60.7	30	1.0
qPCR of virus quantification	ChiLCV-C1/V1q F (GGGCTAAGGTCGAGATGTCC)ChiLCV-q R (CCGGAGGAACTTGAAGAATGG)	62	30	0.2
qPCR of gRNA expression				
i. gRNA-1	IR-spacer3-Top (GGTCAGCCATCCGCACTAATATTAC)gR-qR (GCACCGACTCGGTGCCAC)	64	30	0.096
ii. gRNA-2	IR-spacer6-Top (GATTGCATGGTCCCCCCTATAAACT)gR-qR: (GCACCGACTCGGTGCCAC)	64	30	0.096
iii. gRNA-3	V2/V1-Spacer1-Top (GGTCACATTTCCACGCCCGCCTCGA)gR-qR: (GCACCGACTCGGTGCCAC)	64	30	0.096
iv. gRNA-4	V2/V1-Spacer3-Top (GATTGAGGCCAGAGCATGGGTGAAC)gR-qR: (GCACCGACTCGGTGCCAC)	64	30	0.096
v. gRNA-5	C1/C4-spacer1-Top (GGTCAGGACCCTGAATTGATTGCCT)gR-qR: (GCACCGACTCGGTGCCAC)	64	30	0.25
vi. gRNA-6	C1/C4-spacer2-Top (GATTGTAGCTGATCTTCCATCGACT)gR-qR: (GCACCGACTCGGTGCCAC)	64	30	0.50
qPCR for Cas9	Cas9 qF (GGACCACTTGCTAGAGGAAACTCTC) Cas9 qR (GGAAGGTTCTTATCGAAGTTGGTCATTC)	64	30	0.15
*PP2A* gene primer of *N*. *benthamiana*(reference gene control on qPCR assay for gRNA and Cas9)	PP2A F (GACCCTGATGTTGATGTTCGCT)PP2A R (GAGGGATTTGAAGAGAGATTTC)	59	30	0.2
Actin gene primer of *N*. *benthamiana*(reference gene control on qPCR assay for ChiLCV)	Actin-DNA-qF(AATGATCGGAATGGAAGCTG) Actin-DNA-qR: (TGGTACCACCACTGAGGACA)	61.2	30	0.116

*other parameters of PCR are followed from standard methods. Phusion *Taq* polymerase (Thermo) was used.

### Development of plants expressing multiplexed gRNA-Cas9 modules by using a Golden Gate and Gateway compatible vector toolkit

Plants expressing multiplexed gRNA-Cas9 constructs (duplex and triplex) were generated following the methodology and toolkit established by Lowder et al.[32]. The gRNAs and their complementary sequences were synthesized with the addition of linker sequences at the 5’ or 3’ ends (Table 3). Double-stranded spacer sequences with flanking linkers were produced by annealing the complementary strands of each spacer sequences. The process of developing multiplexed gRNA-Cas9 constructs is as follows: individual double-stranded spacer sequences with flanking linkers were cloned between either the U3 or U6 promoter and the gRNA scaffold sequence of one of the Golden Gate entry plasmids (pYPQ131B, pYPQ132A or pYPQ133B) to generate an individual gRNA clone. One gRNA under the U3 promoter (pYPQ131B derived) and another gRNA under the U6 promoter (pYPQ132A derived) were cloned in tandem into a Gateway entry vector (pYPQ142) via Golden Gate cloning to generate duplexed gRNA clones. Similarly, three Golden Gate entry clones were constructed by cloning the three gRNAs into their respective plasmids (pYPQ131B, pYPQ132A and pYPQ133B). Three gRNA cassettes were subsequently cloned in tandem into pYPQ143 to form a triplexed gRNA Gateway entry clone. A Gateway recombination reaction was performed using a gRNA cassette entry clone (pYPQ142 or pYPQ143), a plant codon-optimized Cas9 (under 35S promoter) entry clone (pYPQ150), and a plant expression Gateway destination vector (pEarleyGate100) to generate duplexed or triplexed gRNA-Cas9 modules. After every step, the resultant clones containing either gRNAs or gRNA-Cas9 expression cassettes were confirmed by sequencing.

**Table 3:** Sequence of double stranded spacer oligos along with linker and the corresponding Golden Gate entry vectors where they were cloned to generate gRNAs.

Selected Spacers	Two strands of spacer oligo sequence with linker (5'-3')*	Entry vector (promoter)	Name of gRNA Constructs
IR-Spacer3	IR-spacer3-Top:ggtcaGCCATCCGCACTAATATTAC	pYPQ131B (U3)	gRNA1
	IR-spacer3-Bot:aaacGTAATATTAGTGCGGATGGCt		
IR-Spacer6	IR-spacer6-Top: gattgCATGGTCCCCCCTATAAACT	pYPQ132A (U6)	gRNA2
	IR-spacer6-Bot: aaacAGTTTATAGGGGGGACCATGc		
V2/V1-Spacer1	V2/V1-Spacer1-Top: ggtcaCATTTCCACGCCCGCCTCGA	pYPQ131B (U3)	gRNA3
	V2/V1-Spacer1-Bot: aaacTCGAGGCGGGCGTGGAAATGt		
V2/V1-Spacer3	V2/V1-Spacer3-Top: gattgAGGCCAGAGCATGGGTGAAC	pYPQ132A (U6)	gRNA4
	V2/V1-Spacer3-Bot: aaacGTTCACCCATGCTCTGGCCTc		
C1/C4-Spacer1	C1/C4-spacer1-Top: ggtcaGGACCCTGAATTGATTGCCT	pYPQ131B (U3) and pYPQ133B (U3)	gRNA5 gRNA5-1
	C1/C4-spacer1-Bot: aaacAGGCAATCAATTCAGGGTCCt		
C1/C4-Spacer2	C1/C4-spacer2-Top: gattgTAGCTGATCTTCCATCGACT	pYPQ132A (U6)	gRNA6
	C1/C4-spacer2-Bot: aaacAGTCGATGGAAGATCAGCTAc		

*Spacer sequences (Top) and its complimentary sequences (Bot) were synthesized with addition of linker (underlined) as required for their cloning into GoldenGate entry vectors. The Top and Bot sequences were annealed to each other to develop double stranded spacer molecule for cloning. The vector has the promoter and gRNA scaffold. After cloning the spacer sequence along with scaffold form the functional gRNAs.

### Plant inoculation

Multiplexed gRNA-Cas9 modules in the pEarleyGate100 vector and the infectious ChiLCV partial tandem dimer in the pCAMBIA2300 vector were individually transformed into *Agrobacterium tumefaciens* strain GV3103 by electroporation. Single colonies from the transformed cells were grown overnight in a selective medium. A fresh culture was prepared from the overnight grown culture by growing it for 3–4 h. The bacterial cells were collected and resuspended in infiltration buffer (10 mM MES [pH 5.7], 10 mM MgCl_2_, and 150 μ M acetosyringone) to obtain OD600 ~0.5. The culture was incubated at room temperature in the dark for 2–4 h and then used to infiltrate the leaves of 4–5 week-old *N*. *benthamiana* plants using a 1.0 mL needleless syringe. Agro-infiltration was performed on three plants for each treatment. Three leaves of each plant were co-inoculated simultaneously with one multiplex gRNA-Cas9 construct and ChiLCV infectious construct. However, to assure that the multiplexed gRNA-Cas9 construct cuts the replicating virus only and not the plasmid backbone of the ChiLCV construct, the inoculation was spatially separated in a way that the multiplexed gRNA-Cas9 module was inoculated onto the portion of lamina adjacent to the petiole, while the ChiLCV infectious construct was inoculated towards the apex of the lamina. It was assumed that after releasing from the plasmid backbone, when the ChiLCV starts replication and moves to the bottom portion of the leaf, it encounters the gRNA-Cas9. Inoculation of ChiLCV infectious construct with an empty gRNA-Cas9 vector backbone served as a positive control, while only the vector backbone inoculated plants served as mock negative controls. At 4 days post-inoculation (dpi), leaf tissues from the gRNA-Cas9 inoculated portion (lamina adjacent to petiole) was harvested and further molecular analysis were performed to determine the virus accumulation and the presence of targeted modifications of the viral sequences. Additionally, symptom development in the newly emerging leaves was recorded. Symptoms were categorized based on two parameters: curling of leaves and stunting of plants. Curling of leaf is graded into the following scale: no visible symptom: 0; flecking, vein thickening and vein twisting: 1; mild puckering and folding of leaves from margin: 2; cupping, rolling and size reduction of leaves: 3. Stunting of plant is compared to that of healthy plant and graded into following scale: no stunting: 0; stunting less than 20%: 1, stunting 20–50%: 2, stunting more than 50%: 3. Only virus inoculated CRISPR-Cas9 non-treated diseased plant will have the highest scale for both categories.

### qPCR assay to detect viral accumulation

Genomic DNA was extracted with cetyl trimethyl ammonium bromide (CTAB) buffer from the gRNA-Cas9 inoculated portion of the *N*. *benthamiana* leaves at 4 dpi. To estimate the effect of genome editing on virus accumulation, quantitative PCR (qPCR) was performed using the SsoAdvanced Universal SYBR Green Supermix and the CFX96 Touch^™^ Real-Time PCR detection system (Bio-Rad, Hercules, CA, USA). The *N*. *benthamiana* Actin gene was used as a reference control. To quantify the ChiLCV from the systemic leaf of promising multiplexed-gRNA inoculated plants, qPCR was also conducted at 20 dpi. The primers used for the qPCR study are listed in Table 2. qPCR assay was performed in three replicates for all the samples.

### Quantitative reverse transcriptase PCR (qRT-PCR) to analyse expression of gRNAs and Cas9

Total RNA was extracted from the gRNA-Cas9 inoculated portion of the *N*. *benthamiana* leaves at 4 dpi using Trizol reagent (Thermo Fisher Scientific). To detect the expression of gRNAs and Cas9 into the inoculated area, qRT-PCR was carried out (Table 1). Briefly, first strand cDNA was synthesized using the iScript^™^ Reverse Transcription Supermix (Bio-Rad). Quantitative RT-PCR was performed using the SsoAdvanced Universal SYBR Green Supermix (Bio-Rad). qRT-PCR was carried out with the spacer-specific primers and the gRNA scaffold primers (Table 2). The *N*. *benthamiana PP2A* gene was used as a reference control [33]. Relative gene expression was calculated using the ΔCT method. qRT-PCR for analyzing the expression of both gRNA and Cas9 was performed in three replicates for all the samples.

### Rolling circle amplification

Virus accumulation was determined in the systemic leaves of promising multiplexed gRNA-Cas9 treated and ChiLCV inoculated plants. Total DNA was isolated 20 dpi and rolling circle amplification (RCA) was carried out using a TempliPhi 100 RCA Kit (GE Healthcare, USA) following the manufacturer’s protocol. DNA from the systemic, symptomatic leaves of mock vector treated, ChiLCV-inoculated plants served as positive control. Each RCA product was separately digested with two enzymes (*Bam*HI and *Xba*I) with unique restriction sites, which were expected to generate full-length virus genome-sized fragment. Digestion products were resolved on 1% agarose gel.

### T7EI assay for detecting mutations

To detect the mutation resulting from DSB repair through the NHEJ pathway, a T7E1 assay was performed as described previously [23]. T7EI assay detect on-target genome editing and estimate editing efficiency using T7 endonuclease I (T7EI), which can cleave a heteroduplex DNA having mutation in any strand. In the T7EI assay, CRISPR-induced mutant gene is amplified by PCR. The PCR products are denatured and reannealed to allow heteroduplex formation between wild-type DNA and CRISPR–mutated DNA. Mutations are then detected using T7EI, which recognizes and cleaves mismatched DNA heteroduplexes. T7EI assay results are analyzed by visualizing cleavage products and full-length amplicons by gel electrophoresis. In this study genomic DNA was isolated from ChiLCV-challenged plants treated either with gRNA-Cas9 or with mock vector (control) samples collected at 4 dpi and was used as the template for PCR using primers ChiLCV-C1/V1q F and ChiLCV-IR R (Table 1). Amplicons of the ChiLCV genomic fragment obtained from those samples were denatured, renatured, and treated with T7EI using the Alt-R^™^ Genome Editing Detection Kit (Integrated DNA Technologies) following the manufacturer’s protocol. Results were evaluated using agarose gel electrophoresis. A control heteroduplex DNA sample provided in the kit served as positive control for the assay.

### TIDE analysis

The ChiLCV genomic portion was amplified from the harvested tissue using two sets of primers ChiLCV-C1/V1q F and ChiLCV IR-R and ChiLCV-IR F and ChiLCV-C1/V1 R using Phusion *Taq* polymerase (Thermo Fisher Scientific). Three amplicons for each samples were sequenced and the sequence was analysed through Tracking of Indels by Decomposition (TIDE) programme (https://tide.nki.nl/) as described previously [34]. TIDE analysis provides rapid and reliable assessment of genome editing efficiencies. It quantifies the rates of NHEJ-mediated repair in an edited sample by decomposing the sequence trace data. It identifies the predominant types of insertions and deletions (indels) in the DNA. Briefly, the virus sequences from gRNA-Cas9 treated and untreated plants were compared for detecting mutation in the region flaking the gRNA target site. All analyses were performed with a default setting.

## Results

### *In vitro* cleavage of ChiLCV DNA by the gRNA-Cas9 RNP complex

**Fig 2:**
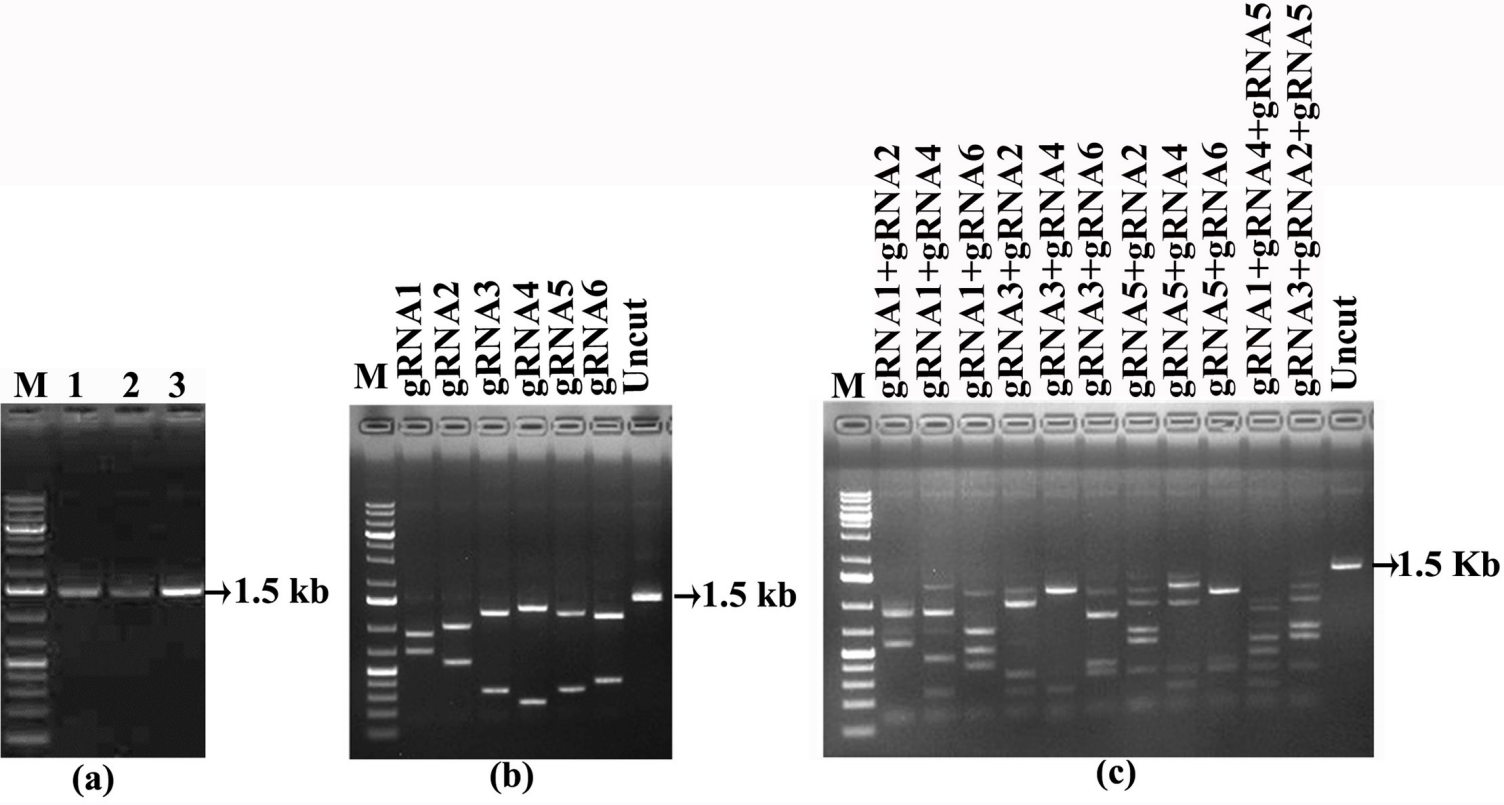
*In vitro* cleavage assay. a) Amplification of the target region from the ChiLCV (lane 1–3 indicates three replicates of same sample) b) *In-vitro* cleavage assay using individual gRNA-Cas9 ribonucleoprotein (RNP) complex. The 1.5-kb viral genomic fragment containing all six target spacer sequences was treated by six individual gRNA-Cas9 RNP complexes. c) *In-vitro* cleavage assay using multiplexed gRNA-Cas9 RNP complex. The 1.5-kb viral genomic fragment containing all six target spacer sequences was treated by duplex or triplex gRNA-Cas9 RNP complexes. Uncut: 1.5-kb viral genomic fragment without the RNP treatment.

Initially six, four and six spacer sequences were identified based on the PAM sequence from IR, V2/V1 and C1/C4 regions of ChiLCV, respectively (Table 1). After off-target analysis and %GC calculation, a total of six spacer sequences, two each from the above three regions in the ChiLCV genome were selected for further analysis (Table 1). To ensure that these spacer sequences could cleave the ChiLCV genome, a 1.5 kb viral genomic fragment containing all six target spacer sequences was amplified from the *N*. *benthamiana* plants inoculated with an infectious clone of ChiLCV (Fig 2A). *In vitro* cleavage assay of the 1.5 kb template using the individual gRNA-Cas9 RNP complex showed efficient cleavage of the template, yielding bands of expected size (Fig 2B). As the main aim was to apply a multiplexed gRNA to cleave the viral genome, we used two or three RNP complexes at a time to see the cleavage products under *in vitro* conditions. The result showed that the multiple RNP complex could cleave the 1.5 kb viral genomic portion into many fragments (Fig 2C). This confirmed that all the spacer sequences that were used to create the RNP complexes could effectively disintegrate the viral genome either individually or in multiplexed modes.

### Multiplexed gRNA-Cas9 complex vectors

In the first step of construct development, six individual gRNA clones were obtained after cloning the double-stranded spacer sequences into a Golden Gate entry vector (Table 3). These gRNAs target the viral genome in the following regions: gRNA1 and gRNA2: IR, gRNA3 and gRNA4: V2/V1 and gRNA5 and gRNA6: C1/C4 regions. In the second step, a total of nine duplex and two triplex gRNA expression clones were generated and used as Gateway entry clones (Table 4). In the third step, same numbers of multiplexed gRNA-Cas9 constructs were cloned into a plant expression vector. The final clone IDs are mentioned in Table 4.

**Table 4:** Duplex and triplex gRNA constructs developed and their impact on disease phenotype.

Sl No.	Multiplex level	gRNA combination	Final clone ID after joining with Cas9	Target region in ChiLCV genome	Disease phenotype grade*
1	Duplex	gRNA1+ gRNA2	gRNA1+2	IR	Leaf curl– 1, Stunting—2
2	Duplex	gRNA1+ gRNA4	gRNA1+4	IR and V2-V1 overlap	Leaf curl– 1, Stunting—2
3	Duplex	gRNA1+ gRNA6	gRNA1+6	IR and C1-C4 overlap	Leaf curl– 2, Stunting—2
4	Duplex	gRNA3+ gRNA2	gRNA3+2	IR and V2-V1 overlap	Leaf curl– 2, Stunting—2
5	Duplex	gRNA3+gRNA4	gRNA3+4	V2-V1 overlap	Leaf curl– 2, Stunting—2
6	Duplex	gRNA3+gRNA6	gRNA3+6	V2-V1 overlap and C1-C4 overlap	Leaf curl– 2, Stunting—2
7	Duplex	gRNA5+gRNA2	gRNA5+2	IR and C1-C4 overlap	Leaf Curl– 0, Stunting—1
8	Duplex	gRNA5+gRNA4	gRNA5+4	V2-V1 overlap and	Leaf curl– 0, Stunting– 0
9	Duplex	gRNA5+gRNA6	gRNA5+6	C1-C4 overlap	Leaf curl– 1, Stunting– 2
1	Triplex	gRNA1+gRNA4+gRNA5-1	gRNA1+4+5	IR, V2-V1 overlap and C1-C4 overlap	Leaf curl– 2, Stunting—2
1	Triplex	gRNA1+gRNA4+gRNA5-1	gRNA3+2+5	IR, V2-V1 overlap and C1-C4 overlap	Leaf curl– 2, Stunting—2

*In mock vector infiltrated ChiLCV inoculated plant (positive control) both the leaf curl and stunting grades were 3, while only mock vector inoculated plant (negative control) both these grades were 0.

### Efficacy of transiently expressed duplex and triplex gRNA-Cas9 complex

**Fig 3:**
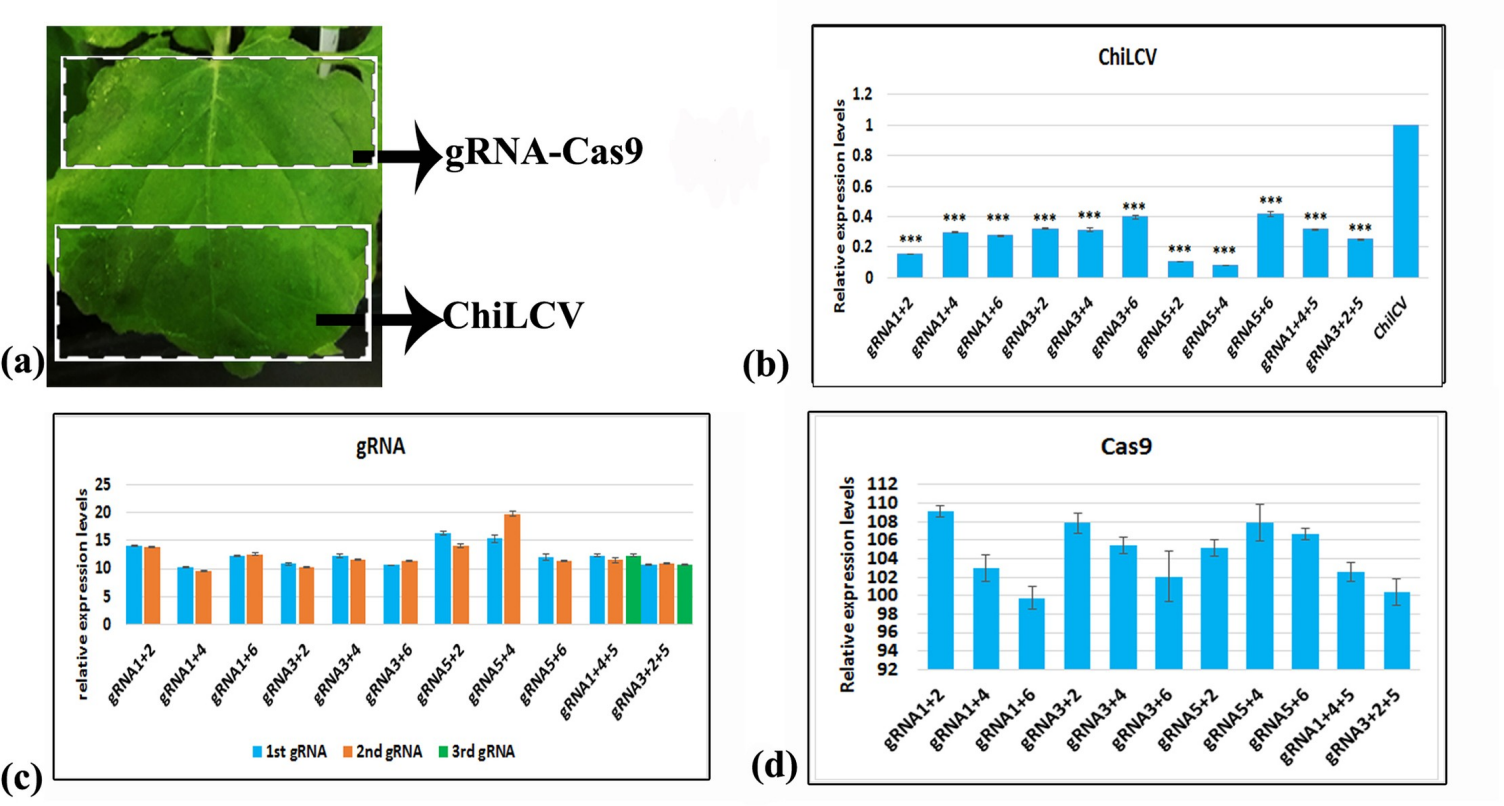
*In planta* expression assay. a) Inoculation positions of gRNA-Cas9 construct and ChiLCV, b) qPCR assay to understand reduction of viral replication, c) qRT-PCR assay for gRNA expression analysis, d) qRT-PCR assay for Cas9 expression analysis. For qPCR and qRT-PCR, three replicates were used for each data point. The error bar denotes SEM. Stars indicate significant difference (student’s unpaired t-test) of ChiLCV expression levels between gRNA-Cas9 treated and non-treated plants (*P< 0.05, **P< 0.01, ***P< 0.001).

**Fig 4:**
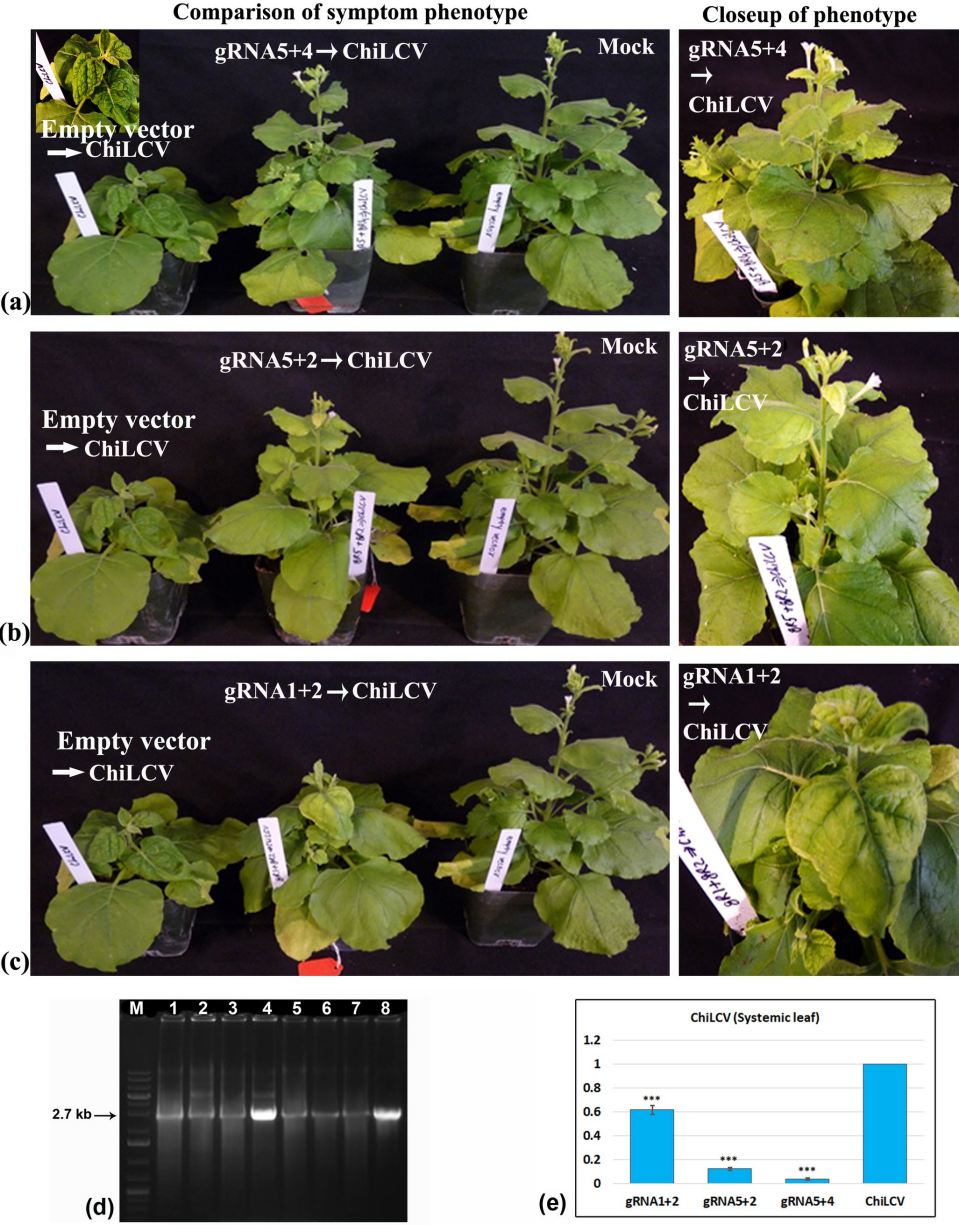
Disease phenotype of *N*. *benthamiana* plants and viral load assay in distal leaves. Test plants were challenged by ChiLCV and infiltrated with three promising gRNA-Cas9 constructs, respectively. Plants treated with mock vector served as negative control while plants treated with mock vector followed by challenged by ChiLCV served as positive control. a) Similar to mock plants, ChiLCV inoculated *N*. *benthamiana* treated with gRNA5+4 did not show any visible leaf curl (grade:0) or stunting (grade:0) symptoms. b) Similar to mock plants, ChiLCV inoculated *N*. *benthamiana* treated with gRNA5+2 did not show visible leaf curl symptom (grade:0), however, a mild stunting (grade:1) phenotype was observed. c) Similar to mock plants, ChiLCV inoculated *N*. *benthamiana* treated with gRNA 1+2 did not show typical leaf curl symptom. However, a mild vein thickening (grade:1) and moderate stunting (grade:2) phenotype was observed. All experiments had three replicates, and they all gave similar results. d) Rolling circle amplification (RCA)-generated ChiLCV genome length fragment (2.7 kb) from systemic leaves of three promising multiplexed gRNA-Cas9 treated and mock vector inoculated plants. RCA product of ChiLCV digested with *BamH*I (Lane 1–4) and *Xba*I (Lane 5–8). Lane 1,5: gRNA1+2 treated, Lane 2,6: gRNA5+2 treated, Lane 3,7: gRNA5+4 treated and Lane 4,8: mock vector treated and ChiLCV inoculated plants e) qPCR assay for analysing viral load from the systemic leaf of plant inoculated with these three gRNA combinations. Three replicates were conducted for each data point. The error bar denotes SEM. Stars indicate significant difference (student’s unpaired t-test) of ChiLCV expression levels between gRNA-Cas9 treated and non-treated plants (*P< 0.05, **P< 0.01, ***P< 0.001).

The efficacy of multiplexed gRNA-Cas9 constructs to cleave the viral DNA under *in planta* condition was determined by measuring the expression level of ChiLCV in the gRNA-Cas9 infiltrated portion by qPCR (Fig 3A). Compared to the mock (vector) inoculated plant, virus accumulation was reduced significantly in plants treated with different combinations of duplex/triplex gRNA-Cas9 (Fig 3B). Among the different gRNA combinations, gRNA5+4 resulted in the lowest level of ChiLCV accumulation followed by gRNA5+2 and gRNA1+2. The triplex gRNA-inoculated plants did not show any added advantage with respect to reduction of ChiLCV accumulation. All combinations had similar levels of gRNA and Cas9 expression (Fig 3C and 3D). In the systemic leaves of all the multiplexed gRNA-Cas9 inoculated plants, appearance of leaf curl symptoms was delayed by five to ten days compared to that in the mock-inoculated plants. The ratings for both leaf curl and stunting phenotypes were lower in all multiplexed gRNA-Cas9 combinations (Table 4). The most promising result with respect to symptom attenuation was observed in plants inoculated with gRNA5+4, where plants did not have any leaf curl symptom (grade 0) nor the plant growth was affected significantly (grade 0) (Fig 4A). The next promising combinations were gRNA5+2 (leaf curl grade:0; stunting grade:1) and gRNA1+2 (leaf curl grade:1; stunting grade:2) (Fig 4B and 4C).

To estimate the viral DNA accumulation in systemic leaves, RCA analysis was performed. The RCA product, when digested with *BamH*I and *Xba*I, two unique restriction sites present in ChiLCV genome, yielded 2.7 kb viral genome length fragments in all the samples. However, concentration of the virus was distinguishably lower in gRNA-Cas9 treated plants (Fig 4D) than that from the mock vector-treated plant. To determine the relative quantification of the viral load in the systemic leaves, qPCR assay of ChiLCV was done, which showed that, in all these three gRNA combinations, there was a significant decrease in virus accumulation (Fig 4E).

### Multiplex gRNA-Cas9 complexes inhibit escape mutant formation

**Fig 5:**
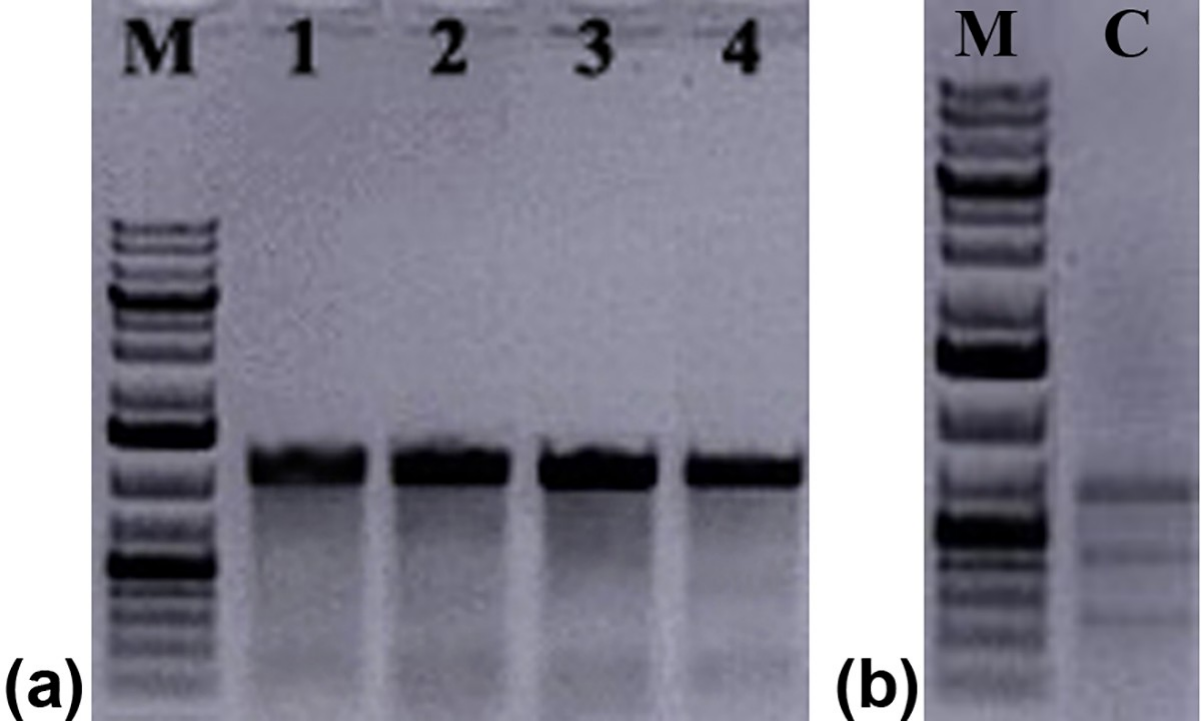
Detection of genome editing by T7E1 assay. a) T7EI assay from gRNA treated ChiLCV challenged plant along with ChiLCV inoculated plant. Lane M, GeneRuler 1kb plus DNA ladder. Samples in lane 1 through 4 were all amplified using primers ChiLCV-C1/V1q F &ChiLCV-IRR and then treated with T7E1. Lane 1, amplicon from mock vector treated ChiLCV inoculated *N*. *benthamiana*. Lane 2, amplicon from gRNA5+4-Cas9 construct treated ChiLCV challenged plants. Lane 3, amplicon from gRNA5+2-Cas9 construct treated ChiLCV challenged plants. Lane 4, amplicon from gRNA1+2-Cas9 construct treated ChiLCV challenged plants. b) Control heteroduplex DNA (C) from the kit showed cleavage of the control heteroduplex by the T7EI.

**Fig 6:**
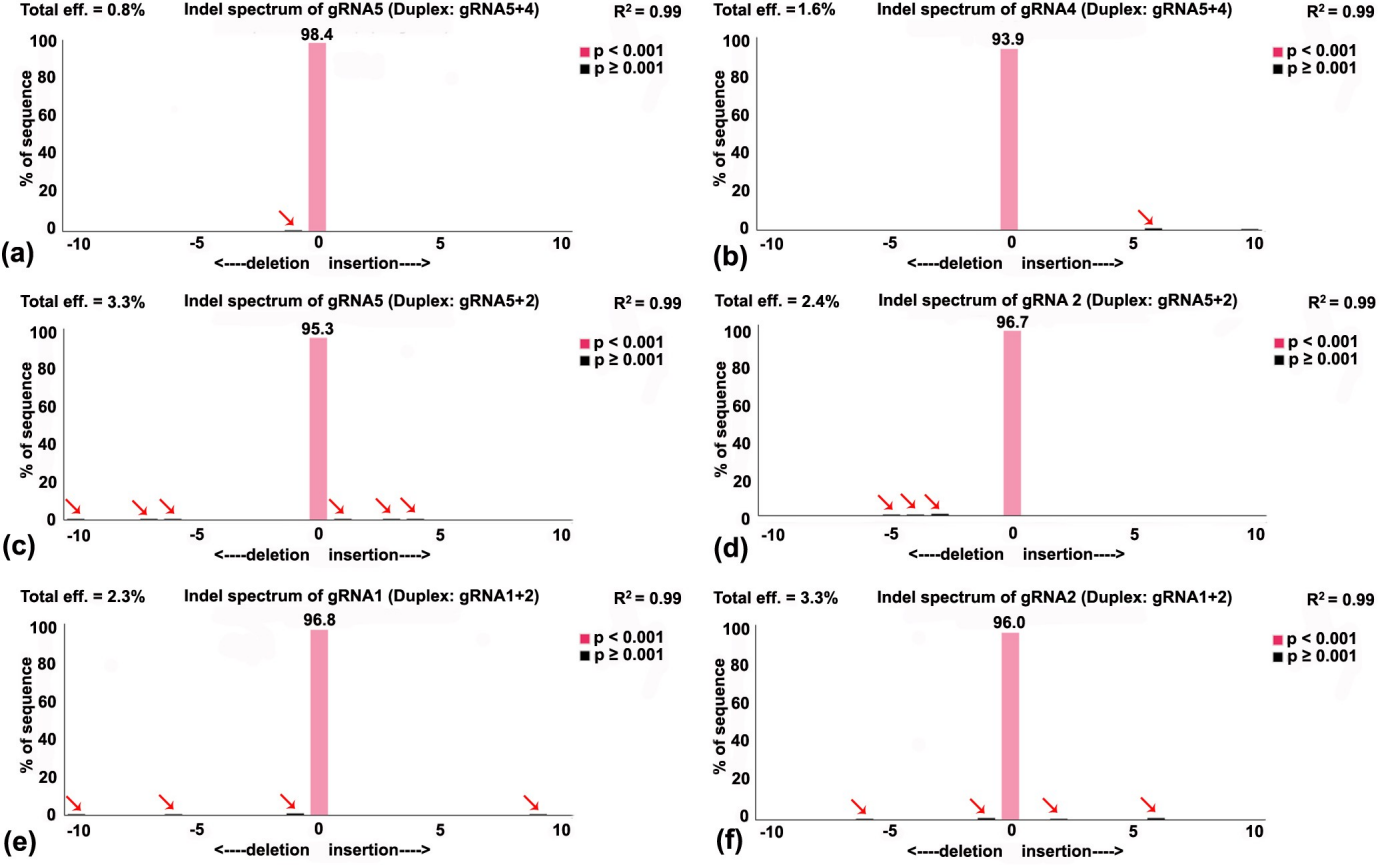
Indel spectrum analysis through TIDE programme. Analyses showed probability of presence of NHEJ generated mutated sequences in upstream or downstream to the PAM motifs in ChiLCV genome after treating with three promising duplex gRNA-Cas9 constructs. Three replicates of the viral genomic sequences from individual plant were identical and hence analysis was done with one sample. Indel spectra generated surrounding the cleavage site of (a) gRNA5 (duplex: gRNA5+4), (b) gRNA4 (duplex: gRNA5+4), (c) gRNA5 (duplex: gRNA5+2), (d) gRNA2 (duplex: gRNA5+2), (e) gRNA1 (duplex: gRNA1+2), (f) gRNA2 (duplex: gRNA1+2). The analysis showed in all combinations no significant escape mutants were present. Arrow indicated insignificant occurrence of mutated sequences surrounding the PAM motif of each gRNA targeting region of ChiLCV.

To evaluate the genome editing, the three promising gRNA-Cas9 combinations mentioned earlier (gRNA 5+4, gRNA 5+2, gRNA 1+2) were tested by T7E1 assay and sequencing. T7E1 assay generated a distinct homoduplex band (wild type virus population) from all three combinations of gRNA-Cas9 but failed to yield recognisable escape mutant fragments even after repeated attempts (Fig 5). The control heteroduplex DNA provided in the kit yielded the expected bands resulting from cleavage of heteroduplex by T7EI enzyme, indicating the effectiveness of the assay. To confirm the T7E1 result, sequencing of the PCR-amplified viral genomic fragment containing the gRNA target revealed presence of only the wild type sequences. To examine the probability of the presence of escape mutants in the PCR amplified product, sequences were analysed using TIDE, which showed that the frequency of the mutation surrounding the PAM sequence is insignificant (Fig 6), further confirming the non-existence of escape mutants in those multiplexed gRNA-Cas9 combinations.

## Discussion

CRISPR-Cas9 is the most widely adopted system for genome editing and has been used successfully in microbes, plants and animals [35]. Recent studies, using geminiviruses, showed that targeting the viral genome at a single site is not a robust strategy to limit viral replication as the escape mutants are generated during the process [24,28]. One of the most interesting features of the CRISPR-Cas9 system is its flexibility to assemble multiple gRNA modules for targeting several genes simultaneously [36,37]. However, there were no known reports of targeting geminiviruses using such multiplex approaches. Baltes et al. [25] showed that when two individual gRNA modules targeting the two regions of same viral genome were inoculated together, they reduce the viral load more effectively than the individual gRNA. In another attempt by Ali et al. [23], a polycistronic tRNA–gRNA based approach was used for simultaneous delivery two gRNAs in a NB-Cas9OE plants. Here, we developed a single cassette containing both Cas9 and combinations of two or three gRNAs and examined if such multiplexed gRNAs specifically targeting more than one gene/region would efficiently reduce the disease development and lower the viral load. It should be easier to use multiplexed gRNAs-Cas9 constructs directly to generate transgenic plants for stable editing.

We first identified spacer sequences from three important regions of the viral genome with an aim to simultaneously target more than a single gene of the virus. These regions are critical for the replication, movement and RNA silencing suppression ability of the virus. Editing of these regions resulted in interfering with the establishment of the infection, reducing viral load and generation of any functional escape mutants. Initially, we demonstrated that synthesized gRNAs, either individually or in combinations, were capable of mediating targeted cleavage of the ChiLCV genome *in vitro*. This finding lead us to believe that similar multiple cleavages must have occurred in the viral genome under our experimental set up *in planta*. Since it was already shown by Ali et al. [24] and Mehta et al. [28] that individual gRNAs could result in escape mutants, and the potential implications for this phenomenon, we did not repeat such individual gRNA strategy, and we focused on expressing multiplxed gRNAs to suppress the generation of escape mutants. Using the multiplex gRNA tool-kit developed by Lowder et al. [32], duplexed and triplexed gRNA-Cas9 constructs were developed and were tested for their ability to reduce the accumulation of ChiLCV genomic DNA as well as leaf curl symptoms in *N*. *benthamiana*. qPCR and qRT-PCR analyses showed that simultaneous targeting of the different coding and non-coding sequences of the ChiLCV resulted in a significant reduction in viral titre (at least a 10-fold reduction for two gRNA combinations). This reduction accompanied an attenuation of viral symptoms in the systemic leaves. It would be interesting to examine the extent of reduction of viral load at later days post inoculation.

Earlier studies showed that the most promising gRNAs were the ones targeting the IR of geminiviruses. In this study, we also obtained severe reduction of viral load with gRNAs targeting the IR region alone (gRNA1+2) or IR region in combination with C1/C4 (gRNA1+6, gRNA5+2) and V2/V1 (gRNA1+4, gRNA3+2). However, the highest level of virus inhibition was observed when gRNAs simultaneously targeting the V2/V1 and C1/C4 were used (gRNA5+4). This construct targeted coat protein and Rep protein of ChiLCV simultaneously leading to maximal deletion of the viral genome, thereby blocking the possibility of generating any escape mutant. To detect any such escape mutants, we analyzed the result of the T7E1 assay through agarose gel electrophoresis. An alternative approach would be use of capillary electrophoresis.

TIDE analysis is a simple process to detect genome editing efficiencies directly from the sequence traces of PCR product thus avoiding tedious cloning and sequencing of all the clones. TIDE can only detect overall indel frequencies, but not nucleotide substitutions or specifically designed indels and thus it can generate *a priori* information on whether the desired mutation(s) which have been introduced are significant or not [38]. Using the sequence traces of three most promising gRNA combinations, TIDE analysis was carried out and it did not detect any significant mutation, indicating after joining of DSB, the mutant virus, if any, was not able to survive. It remains to be determined whether the cloned product from such mixed PCR amplicons will yield any mutant viral sequences. Thus, multiplexed gRNAs provided two benefits: (i) drastic reduction in generation of escape mutants, and (ii) reduced viral load, leading to significant attenuation of viral symptoms. In some cases, such as gRNA5+4, and gRNA5+2), plants were free of any leaf curling.

In order to trigger durable virus resistance using genome editing technology, one would require constitutive and permanent expression of the ribonucleoprotein complex in the host [28]. While this requires producing of stably transformed plants, adopting a transient means, we first set out to know if the multiplex modules could be efficiently applied to inhibit begomoviruses. Similar concern for using multiplexing in a transient assay was also highlighted by Rybicki [29]. Thus, we established the proof of concept by avoiding transgenic approach and by using transient expression to rapidly screen various combinations of gRNAs. We have demonstrated that multiplexed gRNA-Cas9 constructs efficiently inhibited the virus. It is highly likely that this approach will be even more effective, robust, and durable in transgenic plants expressing a higher amount of Cas9.

## Conclusion

In conclusion, we provide here the first proof of concept of multiplexing of gRNA-Cas9 modules to inhibit a begomovirus infection through a transient assay in the model plant *N*. *benthamiana*. We showed such multiplexed CRISPR-Cas9 strategy could reduce virus accumulation significantly without escape mutant formation, which also resulted in significant decrease in disease symptoms. We also identified potential multiplexed-gRNA-Cas9 combinations that were highly effective in imparting resistance. A similar multiplexing strategy could be applicable to other ssDNA viruses of plants.

## Supporting information

S1 File S1_raw_images in pdf. (PDF)

## References

[1] Mansoor S, Briddon RW, Zafar Y, Stanley J. Geminivirus disease complexes: an emerging threat. Trends Plant Sci. 2003; 8: 128–134. 10.1016/S1360-1385(03)00007-4 12663223

[2] Varma A, Mandal B, Singh MK. Globale and spread of whitefly (Bemisiatabaci) transmitted Geminiviruses. In: Thompson W, Editor. The Whitefly, Bemisiatabaci (Homoptera: Aleyrodidae) Interaction with Geminivirus-Infected Host Plants; 2011 pp. 205–292.

[3] Malathi VG, Renukadevi P, Chakraborty S, Biswas KK, Roy A, Sivalingam PN, et al Begomoviruses and Their Satellites Occurring in India: Distribution, Diversity and Pathogenesis. In: Mandal B, Rao G, Baranwal V, Jain R, edtiors. A Century of Plant Virology in India; 2017 pp. 75–177.

[4] Zehra SB, Ahmad A, Sharma A, Sofi S, Lateef A, Bashir Z, et al Chilli Leaf Curl Virus an Emerging Threat to Chilli in India. Int. J. Pure App. Biosci. 2017; 5: 404–414.

[5] Senanayake DMJB, Varma A, Mandal B. Virus–vector Relationships, Host Range, Detection and Sequence Comparison of Chilli leaf curl virus Associated with an Epidemic of Leaf Curl Disease of Chilli in Jodhpur, India. J. Phytopathol. 2012; 160: 146–155.

[6] Zhou X. Advances in understanding begomovirus satellites. Annu. Rev. Phytopathol. 2013; 51: 357–381. 10.1146/annurev-phyto-082712-102234 23915133

[7] Lazarowitz S. Geminiviruses: Genome structure andgene function. Crit. Rev. Plant Sci. 1992; 11: 327–349.

[8] Hanley-Bowdoin L, Settlage SB, Orozco BM, Nagar S, Robertson D. Geminiviruses: models for plant DNA replication, transcription, and cell cycle regulation. Crit. Rev. Biochem. Mol. Biol. 2000; 35: 105–140. 10821479

[9] Fondong VN. Geminivirus protein structure and function. Mol. Plant Pathol. 2013; 14: 635–649. 10.1111/mpp.12032 23615043 PMC6638828

[10] Lapidot M, Legg JP, Wintermantel WM, Polston JE. Management of whitefly-transmitted viruses in open-field production systems. Adv. Virus Res. 2014; 90: 147–206. 10.1016/B978-0-12-801246-8.00003-2 25410102

[11] Vu TV, Choudhury NR, Mukherjee SK. Transgenic tomato plants expressing artificial microRNAs for silencing pre-coat and coat proteins of a begomovirus ToLCNDV, shows tolerance to virus infection. Virus Res. 2013; 172: 35–45. 10.1016/j.virusres.2012.12.008 23276684

[12] Singh A, Taneja J, Dasgupta I, Mukherjee SK. Development of plants resistant to tomato geminiviruses using artificial transacting small interfering RNA. Mol. Plant Pathol. 2015; 16: 724–734. 10.1111/mpp.12229 25512230 PMC6638473

[13] Rojas MR, Macedo MA, Maliano MR, Soto-Aguilar M, Souza JO, Briddon RW, et al World Management of Geminiviruses. Annu. Rev.Phytopathol. 2018; 56: 637–677. 10.1146/annurev-phyto-080615-100327 30149794

[14] Gill U, Scott JW, Shekasteband R, Ogundiwin E, Schuit C, Francis DM, et al Ty-6, a major begomovirus resistance gene on chromosome 10, is effective against Tomato yellow leaf curl virus and Tomato mottle virus. Theor. Appl. Genet. 2019; 132: 1543–1554. 10.1007/s00122-019-03298-0 30758531 PMC6476845

[15] Bonfim K, Faria JC, Nogueira EOPL, Mendes EA, Arago FJL. RNAi mediated resistance to Bean Golden Mosaic Virus in Genetically engineered common bean (Phaseolus vulguris). Mol. Plant Microbe Interact. 2007; 20: 717–726. 10.1094/MPMI-20-6-0717 17555279

[16] Voytas DF, Gao C. Precision genome engineering and agriculture: opportunities and regulatory challenges. PLoS Biol. 2014; 12: e1001877 10.1371/journal.pbio.1001877 24915127 PMC4051594

[17] Barrangou R, Fremaux C, Deveau H, Richards M, Boyaval P, Moineau S, et al CRISPR provides acquired resistance against viruses in prokaryotes. Science 2007; 315: 1709–1712. 10.1126/science.1138140 17379808

[18] Wright AV, Nunez JK, Doudna JA. Biology and applications of CRISPR systems: harnessing nature’s toolbox for genome engineering. Cell 2016; 164: 29–44. 10.1016/j.cell.2015.12.035 26771484

[19] Aouida M, Eid A, Ali Z, Cradick T, Lee C, Deshmukh H, et al Efficient fdCas9 synthetic endonuclease with improved specificity for precise genome engineering. PLoS One 2015; 10: e0133373 10.1371/journal.pone.0133373 26225561 PMC4520497

[20] Piatek A, Mahfouz MM. Targeted genome regulation via synthetic programmable transcriptional regulators. Crit. Rev.Biotechnol. 2016; 37: 429–440. 10.3109/07388551.2016.1165180 27093352

[21] Chaparro-Garcia A, Kamoun S, Nekrasov V. Boosting plant immunity with CRISPR/Cas. Genome Biol. 2015; 16: 254–257. 10.1186/s13059-015-0829-4 26585913 PMC4653885

[22] Hadidi A, Flores R, Candresse T, Barba M. Next-Generation Sequencing and Genome Editing in Plant Virology. Front. Microbiol. 2016; 7: 1325 10.3389/fmicb.2016.01325 27617007 PMC4999435

[23] Ali Z, Abulfaraj A, Idris A, Ali S, Tashkandi M, Mahfouz MM. CRISPR/Cas9-mediated viral interference in plants. Genome Biol. 2015; 16: 238 10.1186/s13059-015-0799-6 26556628 PMC4641396

[24] Ali Z, Ali S, Tashkandi M, Zaidi SS, Mahfouz MM. CRISPR/Cas9-mediated immunity to geminiviruses: differential interference and evasion. Sci. Rep. 2016; 6: 26912 10.1038/srep26912 27225592 PMC4881029

[25] Baltes NJ, Hummel AW, Konecna E, Cegan R, Bruns AN, Bisaro DM, et al Conferring resistance to geminiviruses with the CRISPR–Cas prokaryotic immune system. Nature Plants 2015; 1: 15145. 10.1038/nplants.2015.145 PMC8612103 34824864

[26] Ji X, Zhang H, Zhang Y, Wang Y, Gao C. Establishing a CRISPR–Cas-like immune system conferring DNA virus resistance in plants. Nature Plants 2015; 1: 15144 10.1038/nplants.2015.144 27251395

[27] Liu HJ, Soyars CL, Li JH, Fei QL, He GJ, Fei Q, et al CRISPR/Cas9-mediated resistance to cauliflower mosaic virus. Plant Direct. 2018; 2: e00047 10.1002/pld3.47 31245713 PMC6508564

[28] Mehta D, Stürchler A, Anjanappa RB, Zaidi SS, Hirsch-Hoffmann M, Gruissem W, et al Linking CRISPR-Cas9 interference in cassava to the evolution of editing-resistant geminiviruses. Genome Biol. 2019; 20: 80 10.1186/s13059-019-1678-3 31018865 PMC6482539

[29] Rybicki EP. CRISPR–Cas9 strikes out in cassava. Nature Biotechnol. 2019; 37: 727–728. 31197230 10.1038/s41587-019-0169-0

[30] Zaidi SSA, Tashkandi M, Mahfouz MM. Engineering molecular immunity against plant viruses. Prog. Mol. Biol. Transl. 2017; 149: 167–186. 10.1016/bs.pmbts.2017.03.009 28712496

[31] Shilpi S, Kumar A, Biswas S, Roy A, Mandal B. A recombinant Tobacco curly shoot virus causes leaf curl disease in tomato in a north-eastern state of India and has potentiality to trans-replicate a non-cognate betasatellite. Virus Genes 2015; 50: 87–96. 10.1007/s11262-014-1141-1 25410052

[32] Lowder LG, Zhang D, Baltes NJ, Paul JW, Tang X, Zheng X, et al A CRISPR/Cas9 toolbox for multiplexed plant genome editing and transcriptional regulation. Plant Physiol. 2015; 169: 971–985. 10.1104/pp.15.00636 26297141 PMC4587453

[33] Liu D, Shi L, Han C, Yu J, Li D, Zhang Y. Validation of reference genes for gene expression studies in virus-infected Nicotiana benthamiana using quantitative real-time PCR. PLoS One 2012; 7: e46451 10.1371/journal.pone.0046451 23029521 PMC3460881

[34] Brinkman EK, Chen T, Amendola M, van Steensel B. Easy quantitative assessment of genome editing by sequence trace decomposition. Nucleic Acids Rec. 2014; 42: e168 10.1093/nar/gku936 25300484 PMC4267669

[35] Hsu PD, Lander ES, Zhang F. Development and applications of CRISPR-Cas9 for genome engineering. Cell 2014; 157: 1262–1278. 10.1016/j.cell.2014.05.010 24906146 PMC4343198

[36] Xing HL, Dong L, Wang ZP, Zhang HY, Han CY, Liu B, et al A CRISPR/Cas9 tool kit for multiplex genome editing in plants. BMC PlantBiol. 2014; 14: 327–339. 10.1186/s12870-014-0327-y PMC4262988 25432517

[37] Xie K, Minkenberg B, Yang Y. Boosting CRISPR/Cas9 multiplex editing capability with the endogenoust RNA-processing system. Proc. Natl. Acad. Sci. U.S.A. 2015; 112: 3570–3575. 10.1073/pnas.1420294112 25733849 PMC4371917

[38] Brinkman EK, van Steensel B. Rapid Quantitative Evaluation of CRISPR Genome Editing by TIDE and TIDER In: Luo Y. (eds) CRISPR Gene Editing. Methods in Molecular Biology, vol 1961 Humana Press, New York, NY 2019. 10.1007/978-1-4939-9170-9_3 30912038

