# Supplementary figures and images for: Multiplexed editing of a begomovirus genome restricts escape mutant formation and disease development
Source: PLoS One. 2019 Oct 23;14(10):e0223765. doi: 10.1371/journal.pone.0223765 (PMC6808502; doi:10.1371/journal.pone.0223765)

Image File for Gels

Fig 2 a

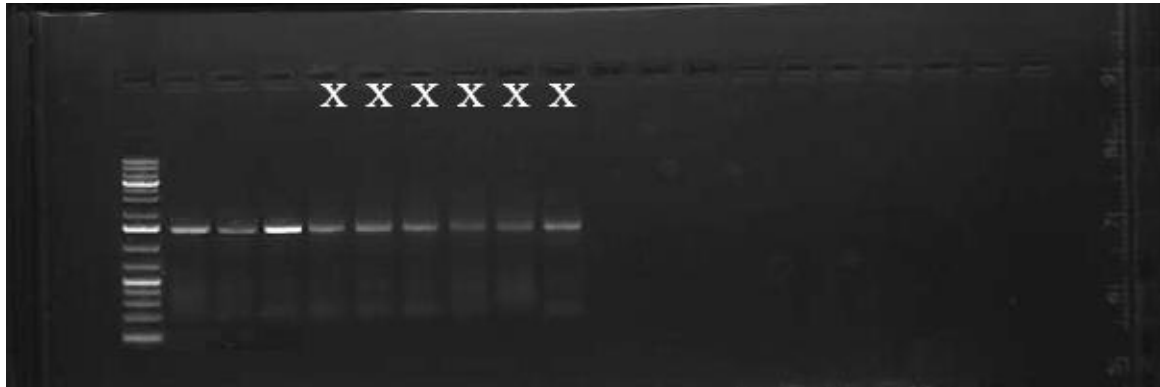

Fig 2 b

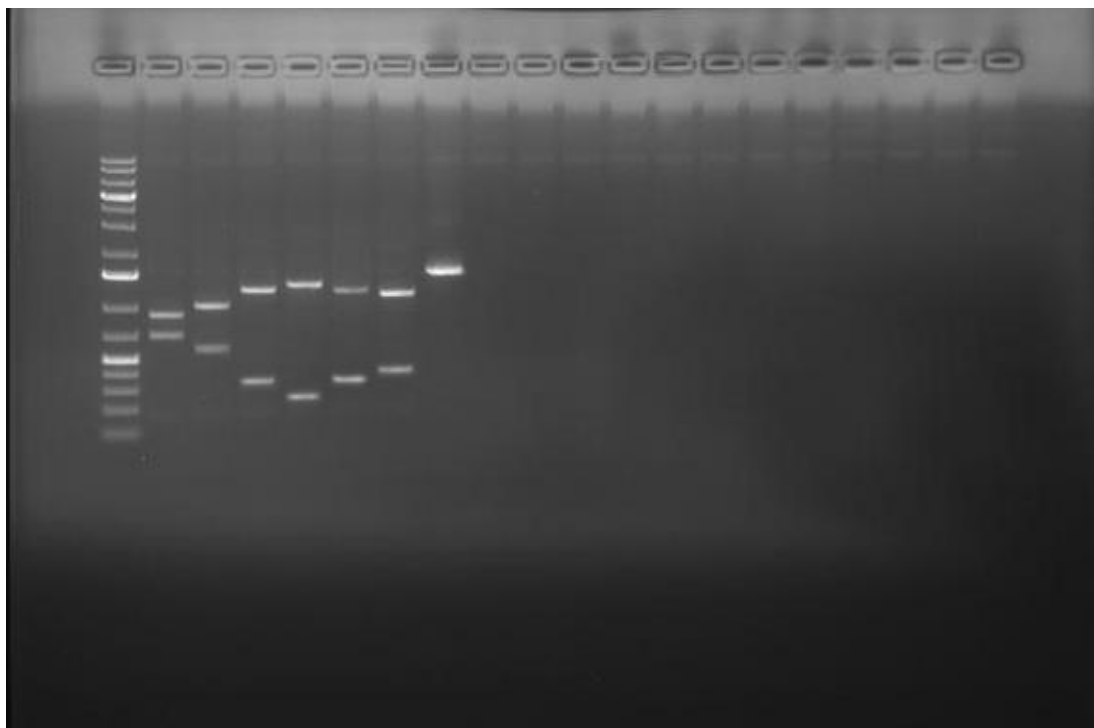

Fig 2 c

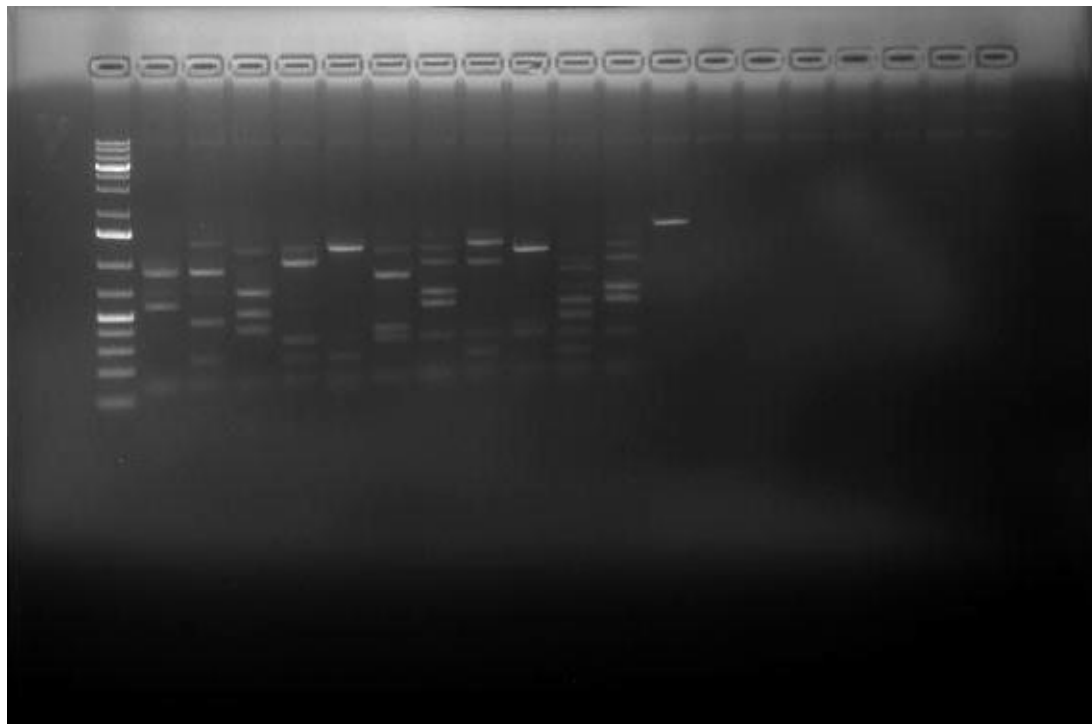

Fig 4 d

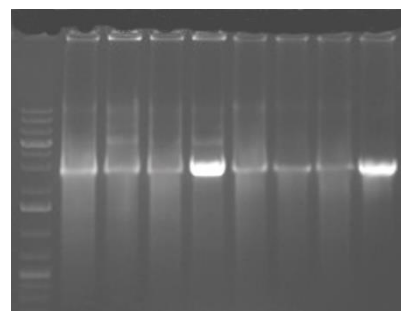

Fig 5 a

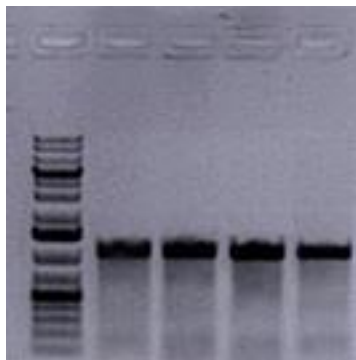

Fig 5 b

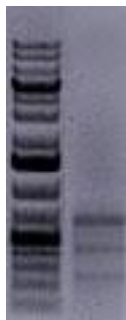

Supplement: S1 File — (PDF) [file pone.0223765.s001.pdf]
